# Effects of Long Noncoding RNA HOXA-AS2 on the Proliferation and Migration of Gallbladder Cancer Cells

**DOI:** 10.1155/2022/6051512

**Published:** 2022-10-17

**Authors:** Peng Zhang, Luhao Liu, Weiting Zhang, Jiali Fang, Guanghui Li, Lei Zhang, Jiali Li, Xuanying Deng, Junjie Ma, Kun Li, Zheng Chen

**Affiliations:** ^1^Organ Transplant Center, The Second Affiliated Hospital of Guangzhou Medical University, Guangzhou 511447, China; ^2^Department of Hepatobiliary Surgery, The Second People's Hospital of Guiyang, Guiyang 550023, China

## Abstract

To explore the function and mechanism of lncRNA HOXA-AS2 in cancer-associated fibroblasts (CAFs)-derived exosomes in gallbladder cancer metastasis, and provide new research targets for the treatment of gallbladder cancer. At the same time, in order to clarify the early predictive value of lncRNA HOXA-AS2 for gallbladder cancer metastasis, and to provide a theoretical basis for clinical individualized treatment of gallbladder cancer. *Methods*. In our previous work, we used TCGA database analysis to find that lncRNA HOXA-AS2 was highly expressed in gallbladder cancer tissues compared with normal tissues. In this study, the expression levels of HOXA-AS2 in gallbladder cancer cell lines and control cells were first verified by QPCR and Western blot methods. Then, lentiviral tools were used to construct knockdown vectors (RNAi#1, RNAi#2) and negative control vectors targeting two different sites of HOXA-AS2, and the vectors were transfected into NOZ and OCUG-1 cells, respectively. Real-time PCR was used to detect knockdown efficiency. Then, the effects of silencing HOXA-AS2 on the proliferation, cell viability, cell migration, and invasion ability of gallbladder cancer cells were detected by MTT, plate cloning assay, Transwell migration chamber assay, and Transwell invasion chamber assay. Finally, the interaction between HOXA-AS2 and miR-6867 and the 3′UTR of YAP1 protein was detected by luciferase reporter gene. The results showed that the expression level of HOXA-AS2 in gallbladder cancer cell lines was higher than that in control cells. The expression of HOXA-AS2 in gallbladder carcinoma tissues was significantly higher than that in adjacent tissues (*p* < 0.05). After successful knockout of HOXA-AS2 by lentiviral transfection, the expression of HOXA-AS2 in gallbladder cancer cell lines was significantly decreased. Through cell proliferation and plate clone detection, it was found that silencing HOXA-AS2 inhibited cell proliferation and invasion. Through software prediction and fluorescein reporter gene detection, it was found that HOXA-AS2 has a binding site with miR-6867, and the two are negatively correlated, that is, the expression of miR-6867 is enhanced after the expression of HOXA-AS2 is downregulated. And the 3′UTR of YAP1 protein in the Hippo signaling pathway binds to miR-6867. Therefore, HOXA-AS2 may affect the expression of YAP1 protein by regulating miR-6867, thereby inhibiting the Hippo signaling pathway and promoting the proliferation and metastasis of gallbladder cancer cells. HOXA-AS2 is abnormally expressed in gallbladder cancer cells. HOXA-AS2 may promote the migration and invasion of gallbladder cancer cells by regulating the Hippo signaling pathway through miR-6867. HOXA-AS2 may serve as a potential diagnostic and therapeutic target for gallbladder cancer in clinic.

## 1. Introduction

Clinically, gallbladder cancer (GBC), as one of the nauseating tumors, has a very high mortality rate despite its low incidence [1], usually found in the biliary system [2, 3]. However, due to the limited potential for curative resection and its resistance to chemotherapeutic agents, gallbladder carcinoma is an aggressive malignancy with high mortality [4]. Finding therapeutic targets for gallbladder cancer is an important process to prolong the survival of patients with gallbladder cancer. Local invasion and distant metastasis are important biological features of gallbladder cancer. The development of effective molecular targets plays an important role in inhibiting the metastasis of gallbladder cancer. Many previous studies have found that miRNAs are involved in the metastatic process of gallbladder cancer. Bao et al. found that miR-101 inhibits the metastasis of gallbladder cancer [5]. MicroRNA-135a [6] and miR-20 [7] have been shown to be closely related to gallbladder cancer metastasis.

In the field of oncology research, a number of studies in recent years have demonstrated that lncRNAs are involved in the formation and development of tumors [8–10]. lncRNAs in the invasion of gallbladder cancer has so far been unclear. On the basis of previous studies, screening gallbladder cancer cell lines with different metastatic characteristics and searching for differentially expressed genes/lncRNAs by sequencing is a more effective research method. Through this experimental method, Wang et al. successfully demonstrated that CLIC1 promotes the migration and invasion of gallbladder cancer cells [11]. Related studies have shown that lnc-H19 promotes gallbladder cancer metastasis by regulating EMT [12]. lnc-CCAT1 promotes gallbladder cancer metastasis by negatively regulating miR-218-5p [13]. Therefore, it is necessary to screen lncRNAs related to gallbladder cancer metastasis by lncRNA chip. In our previous work, we collected tumor tissues from patients with gallbladder cancer, isolated CAFs-derived exosomes, and used lncRNA microarray chip and TargetScan software to analyze the differential lncRNAs related to metastasis. Finally, an lncRNA with significant differential expression was screened out, namely, lncRNA HOXA-AS2. The lncRNA HOXA-AS2 is an unknown lncRNA, and its biological function and mechanism of action are unclear. Therefore, lncRNA HOXA-AS2-specific siRNAs targeting different targets which were selected for a series of experimental studies to study their effects invasion of gallbladder cancer cells at the cellular level and to explore new targets for molecular targeted therapy of gallbladder cancer.

## 2. Materials and Methods

### 2.1. Cells, Tissue Samples, and Clinicopathological Data

Human gallbladder cancer cell lines: GEC, SGC-996, EH-GB, NOZ, GBC-SD, and OCUG-1. Clinical tissue samples from 15 patients with gallbladder cancer who underwent cholecystectomy for gallbladder cancer in the Second Affliated Hospital of Guangzhou Medical University and Zhujiang Hospital of Southern Medical University from May 2019 to March 2021 were collected, including cancer tissues and normal paracancerous tissues of different TNM stages. Collected clinical tissue samples were kept in liquid nitrogen until total RNA extraction.

### 2.2. Real-Time PCR

For the extraction of total RNA from gallbladder cancer cell lines and clinical gallbladder cancer tissue samples, the specific steps refer to previous studies [14]. The extracted total RNA was synthesized into cDNA using a reverse transcription system kit (Thermo Fisher Scientific) as a reaction template for real-time fluorescence quantitative PCR. The content of lncRNA HOXA-AS2 was detected using a fluorescence quantitative PCR kit (Applied Biosystems, USA). Refer to previous studies for specific steps [15].

### 2.3. Cell MTT Assay

The NOZ cells and OCUG-1 cells in logarithmic growth phase in which the HOXA-AS2 gene was knocked out were collected and counted. Select an appropriate cell density for passage in a 96-well plate (about 5,000 cells per well), set 3 parallel wells, and take out a well every 48 hours. Add preprepared MTT solution (Aladdin, Shanghai) and DESO solution.

### 2.4. Cell Clone-Formation Assay

The gallbladder cancer cell lines before and after the knockout of the HOXA-AS2 gene were selected for cell clone formation experiments, including, trypsinizing and counting cells in logarithmic growth phase, and inoculating cells with appropriate density in 6-well plates (each well). Seed about 500-2000 cells, mix well, and set 3 parallel wells. Finally, observe under an inverted microscope, count and take pictures, and make statistics of the results.

### 2.5. Cell Transwell Migration and Invasion Assay

Select NOZ and OCUG-1 cells successfully transfected with RNAi#1, RNAi#2, and NC (and the cells are in logarithmic growth phase), and use serum-free cell culture medium to culture the cells overnight before the experiment to reduce the effect of serum on the experiment. Cells were then trypsinized, washed 3 times with serum-free medium, counted, and made into suspension. Add the cell suspension to the Transwell chamber and incubate the cells with serum-free medium. PBS solution was used to wash the cells that did not invade the upper layer and were observed, photographed, and counted under a microscope (Zeiss, Germany). Data processing and result analysis are then carried out. The specific steps of related experiments refer to previous studies [16].

### 2.6. Preparation of Gallbladder Cancer Cell Lines with Reduced HOXA-AS2 Expression Mediated by Lentivirus Infection

Select freshly grown 30% monolayer cells as transfected cells for future use. The transfection groups are as follows: blank group NOZ cells, OCUG-1 cells (without any treatment); negative control NC (transfected with Scramble siRNA); and experimental group: against specific valid sequences of lncRNA HOXA-AS2 targeting two different target sites and GV112 lentiviral integration plasmids (RNAi#1, RNAi#2). Change the cell culture medium to serum-free medium before infection, and add HOXA-AS2 to interfere with lentivirus sh-RNAi#1, sh-RNAi#2, and negative control lentivirus sh-Ctrl according to 10MOI (multiplicity of infection). To infect cells, add a certain amount of polybrene solution to the cell culture medium to improve the efficiency of virus infection. Normal cell passaging was performed after cells were confluent. On the 4th day after infection, the virus infection of cells was checked with an inverted fluorescence microscope, and finally NOZ and OCUG-1 cells with knockdown of HOXA-AS2 were obtained and analyzed by qRT-PCR. Interference efficiency at different sites in HOXA-AS2 was obtained.

### 2.7. Western Blot

The total protein extraction kit (Teyebio, Shanghai, China) was used to lyse and extract tissue proteins and cellular proteins. The specific experimental operation can refer to the previous research [17]. The protein concentration was subsequently determined using the BCA method (Teyebio, Shanghai, China). After the denatured protein was separated by gel running, the resultant was blotted onto a polyvinylidene fluoride membrane. The entire transfer system was placed in an ice-water mixture, and the membrane was transferred for about an hour under the conditions of 100 V, 400 mA. This was followed by overnight incubation with 5% nonfat dry milk in blocking solution. Use the desired antibody as the primary antibody to incubate the blocked PVDF membrane according to the instructions, and add an appropriate amount of secondary antibody and incubate with shaking at room temperature. A development kit (Teyebio, Shanghai, China) visualized the bands. The antibodies used are as follows: CyclinD1, p21, MMP9, snail, YAP1, p-YAP, TAZ, p-TAZ, GAPDH antibodies (1 : 2000, Abcam), and HRP labeled IgG antibody (1 : 10000, Cell Signaling Technology).

### 2.8. Luciferase Reporter Gene Assay

Wild and mutant HOXA-AS2/YAP1 was cotransfected with miR-6867-5p mimic/NC into HEK-293 T cells. Then luciferase activity was measured on a luciferase reporter system (Promega) using a dual-luciferase reporter gene detection kit (Beyotime, Shanghai, China).

### 2.9. Statistical Analysis

Statistical software SPSS22.0 was used for data analysis. All data were repeated at least 3 times. The two-tailed student's *t*-test was used to assess the difference between the two groups, and the level of statistical difference was expressed as *p* value: ^∗^, *p* value <0.05.

## 3. Results

### 3.1. HOXA-AS2 Expression Pattern in Gallbladder Carcinoma

**Figure 1:**
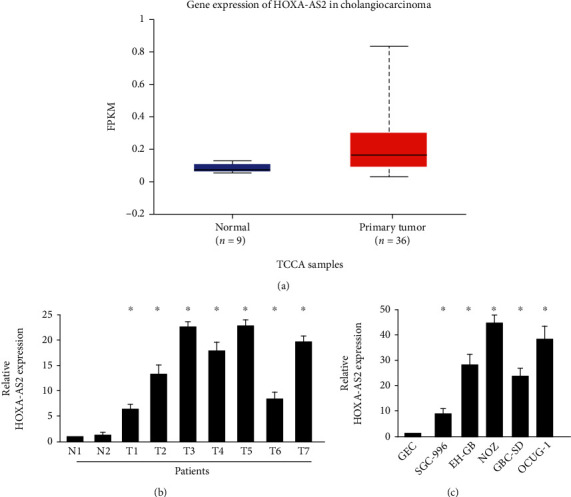
Expression of HOXA-AS2 in clinical tissue samples and cell lines of patients with gallbladder cancer. (a) Shows the high expression of HOXA-AS2 in gallbladder cancer tissues and low expression in normal tissues from the analysis of TCGA database. (b, c) The expression of HOXA-AS2 in clinical tissues and cell line samples of gallbladder cancer, respectively.

In the TCGA database, the expression of HOXA-AS2 was analyzed in normal tissues and gallbladder cancer tissues, and it was found that HOXA-AS2 was abnormally expressed in gallbladder cancer tissues (Figure 1(a)). Analyze the level of lncRNA HOXA-AS2 in clinical tissue samples of different stages of gallbladder cancer. The expression of HOXA-AS2 in the clinical tissues of different stages of gallbladder cancer was higher than that in the corresponding normal tissues (Figure 1(b)). In addition, we further detected the expression of lncRNA HOXA-AS2 in hepatoma cells. As shown in Figure 1(c), the level of HOXA-AS2 in normal gallbladder cell GECs was used as a reference. HOXA-AS2 was highly expressed in multiple gallbladder cancer cell lines.

### 3.2. Effects of Knockdown of HOXA-AS2 on Viability, Proliferation, Migration, and Invasion of Gallbladder Cancer Cells

**Figure 2:**
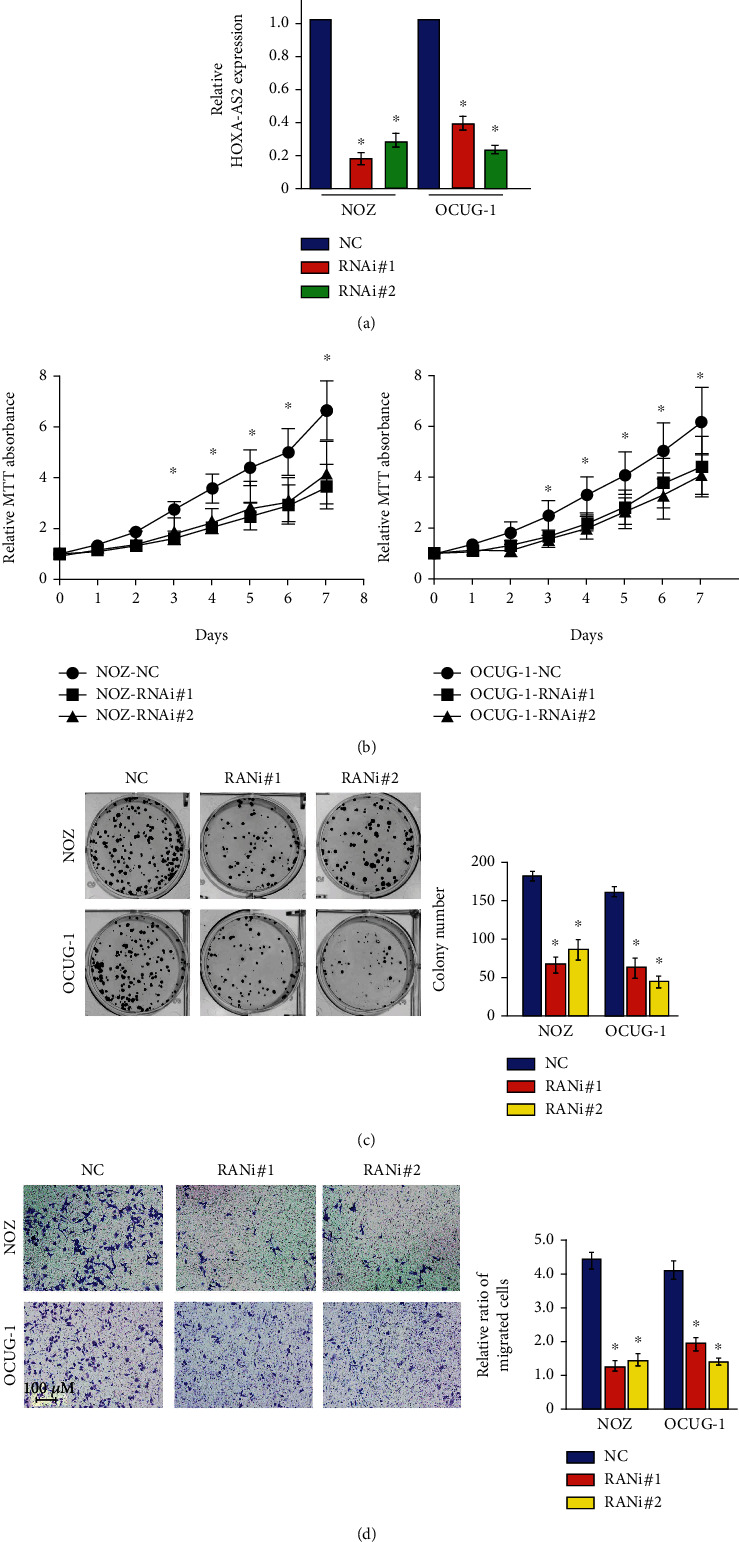
Effects of HOXA-AS2 knockout on proliferation, migration, and invasion of gallbladder cancer cells. (a) Real-time PCR results show that the expression of HOXA-AS2 in the knockdown group is significantly decreased. (b) MTT detects the change of cell viability after silencing HOXA-AS2 cells. (c) Clone formation detects the silence of HOXA-AS2 cells. Cell proliferation ability after AS2. (d) Transwell migration and invasion assay to detect changes in cell migration and invasion ability after HOXA-AS2 silencing.

NOZ and OCUG-1 cells were transfected with specific shRNAs (RNAi#1, RNAi#2) targeting two different sites of lncRNA HOXA-AS2 carried by lentiviral tools, 96 hours after transfection, the expression level of RNAi#2 was only 2.6% of the Lv-shCon (NC) group (Figure 2(a)). The above results suggested that Lv-shHOXA-AS2 specifically knocked down HOXA-AS2 in NOZ and OCUG-1 cells. MTT cell viability assays and cell colony formation experiments were performed. As shown in Figure 2(b), we found that the absorbance at 490 nm of NOZ and OCUG-1 cells infected with sh-HOXA-AS2 lentivirus was significantly lower than that of cells infected with sh-Ctrl lentivirus, indicating that knockdown of SNHG16 inhibited viability of NOZ and OCUG-1 cells. Clonogenic assays showed decreased cell growth in NOZ and OCUG-1 cells knocked down HOXA-AS2 (Figure 2(c)). The results of in vitro proliferation experiments showed that the level of HOXA-AS2 was downregulated in NOZ and OCUG-1 cells and inhibited cell proliferation. In addition, in the Transwell migration and invasion experiments, we found that under the premise of maintaining the same initial cell number, after 48 hours of cell culture, the NOZ and OCUG-1 cells in the HOXA-AS2 expression-decreased group showed reduced migration and invasion cells (Figure 2(d)), suggesting that the mutation of HOXA-AS2 inhibits the migration and invasion of cancer cells in vitro.

### 3.3. Knockdown of lnc-HOXA-AS2 Reduces the Expression of Transcription Factors Associated with Proliferation and Metastasis in NOZ and OCUG-1 Cells

**Figure 3:**
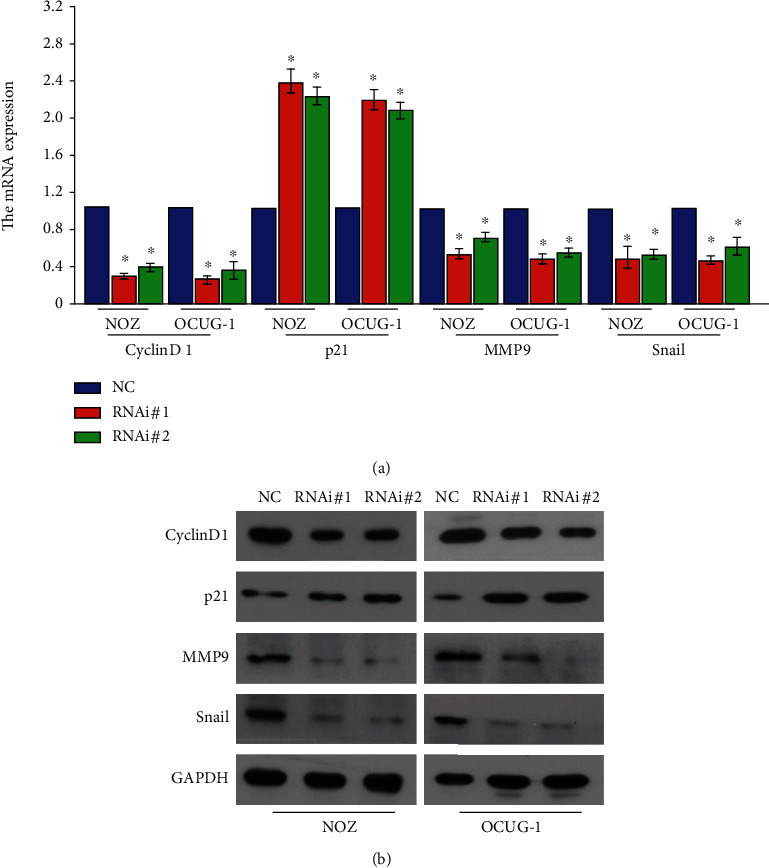
Expression of related regulatory factors affecting the cycle of gallbladder cancer cells after knockout of HOXA-AS2. QPCR of (a) Western blot. (b) Detection of knockdown of HOXA-AS2 after knockdown of HOXA-AS2 significantly decreased the expression of key regulators related to cell proliferation, metastasis, and invasion.

The expression of key cell cycle-related regulators CyclinD1 and P21 proteins was detected by real-time PCR and Western blot (Figures 3(a) and 3(b)). The expression of CyclinD1 protein was found to be decreased in the RNAi#1 and RNAi#2 groups. The levels of MMP9 and Snail, which affect cell migration and invasion were subsequently detected, and the levels of MMP9 and Snail were significantly reduced after silencing lnc-HOXA-AS2 (Figures 3(a) and 3(b)).

### 3.4. miR-6867-5p Is a Downstream Target of lncRNA HOXA-AS2

**Figure 4:**
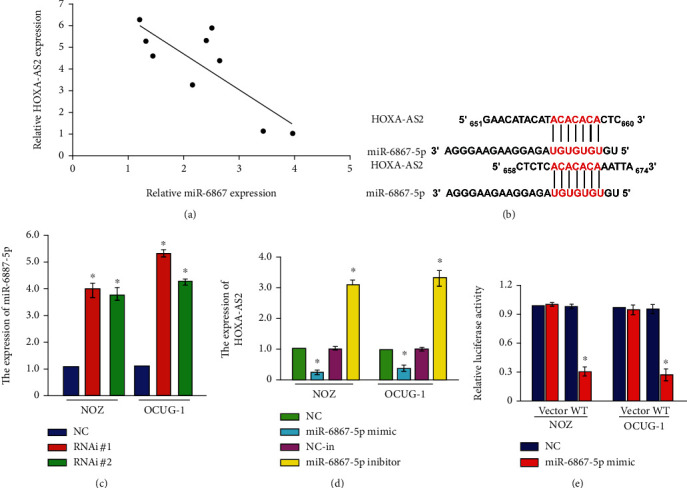
Regulation of miR-6867-5p by HOXA-AS2. (a) RT-qPCR detection of the correlation between HOXA-AS2 and miR-6867-5p expression. (b) Targets can predict the binding site prediction of HOXA-AS2 and miR-6867-5p. (c) RT-qPCR to detect the expression level of miR-6867 after HOXA-AS2 silencing in NOZ and OCUG-1 cells. (d) RT-qPCR is used to detect the overexpression of miR-6867 in NOZ and OCUG-1 cells and the expression of HOXA-AS2 after expression inhibition expression level; luciferase activity to detect the target regulation of HOXA-AS2 and miR-6867.

RNA from clinical tissue samples of gallbladder carcinoma was detected using RT-PCR, and the expression of HOXA-AS2 was found to be inversely correlated with MiR-6867-5p (Figure 4(a)). Subsequently, the results were analyzed by TargetScan software, and it was found that HOXA-AS2 and miR-6867 have the same binding site (Figure 4(b)). MiR-6867-5p was significantly increased in NOZ and OCUG-1 cells in RNAi#1 and RNAi#2 group which HOXA-AS2 was knocked down (Figure 4(c)). HOXA-AS2 expression was decreased after overexpression of miR-6867 in NOZ and OCUG-1 cells and increased upon addition of miR-6867 inhibitor (Figure 4(d)). These experimental results indicated that HOXA-AS2 was inversely correlated with the expression of miR-6867-5p in related cell lines. These further support the idea that miR-6867-5p is the target of lncRNA HOXA-AS2.

### 3.5. lncRNA HOXA-AS2 Affects the Occurrence and Development of Gallbladder Cancer Cells by Regulating miR-6867-5p/YAP1

**Figure 5:**
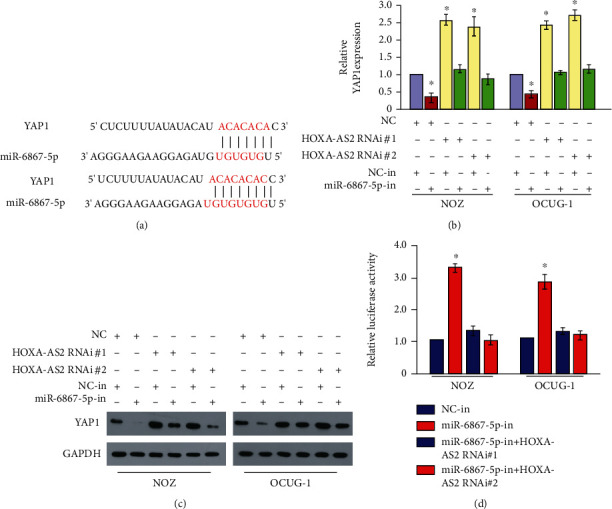
YAP1-related regulatory effects of HOXA-AS2 and miR-6867-5p. (a) Targets can software analyze the prediction of YAP1 3'UTR binding to miR-6867-5p targets. (b) RT-qPCR detects HOXA-AS2 and miR-6867 regulates the expression of YAP. (c) Western blot detection of HOXA-AS2 and miR-6867 regulate the expression of YAP. (d) Luciferase activity detection of HOXA-AS2 and miR-6867 regulation of YAP 3'UTR luciferase activity changes.

We performed analysis using TargetScan prediction software and found that YAP1 was a target of miR-6867-5p (Figure 5(a)). Subsequently, by RT-qPCR and Western blot detection, the level of YAP1 was increased in the RNAi#1/RNAi#2 group and miR-6867-5p overexpression group. RNAi#1/RNAi#2 group and miR-6867-5p significantly decreased in the inhibitor group (Figures 5(b) and 5(c)). As lncRNA HOXA-AS2 was not silenced resulting in upregulation of miR-6867-5p, this may further upregulate YAP1 levels. Furthermore, we found that the luciferase activity was significantly reduced in the HOXA-AS2-miR-6867-5p group at two specific sites (Figure 5(d)). All the results demonstrate a consistent axis of regulatory relationship between lncRNA HOXA-AS2-miR-6867-5p–YAP1.

**Figure 6:**
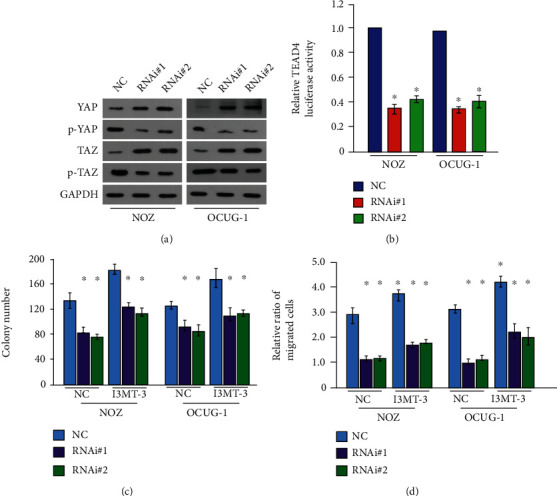
HOXA-AS2 affects the proliferation and invasion of gallbladder cancer cells through the regulation of YAP1 on the Hippo pathway. (a) Western blot detection of YAP and TAZ phosphorylation activation in Hippo signaling pathway after HOXA-AS2 silencing. (b) Luciferase activity detection of TEAD transcription factor activation after HOXA-AS2 downregulation (c). Clonal formation assay to detect Hippo pathway agonist-stimulation cell proliferation ability. (d) Transwell assay to detect cell invasion ability after Hippo pathway agonist stimulation.

The transcriptional coactivator YAP/TAZ in the Hippo signaling pathway loses its transcriptional activity when phosphorylated, and YAP/TAZ itself is a transcriptional coactivator that cannot bind DNA, so it needs to be combined with other transcription factors such as TEAD1-4, coinitiated the transcription of downstream genes. Therefore, we detected the phosphorylation and activation of YAP and TAZ after HOXA-AS2 knockdown by Western blot. The phosphorylation of YAP was significantly reduced in RNAi#1\RNAi#2 gallbladder cancer cells (Figure 6(a)). At the same time, the results of luciferase activity detection showed that the activities of TEAD1-4 transcription factors that interacted with YAP also decreased correspondingly (Figure 6(b)), indicate that silencing of HOXA-AS2 affects the expression of Hippo signaling pathway-related regulators. In addition, adding an agonist of the Hippo signaling pathway to HOXA-AS2 knockdown gallbladder cancer cells, and through cell cloning and invasion experiments found that the increased expression of Hippo signaling pathway-related regulators inhibited the proliferation and invasion of gallbladder cancer cells (Figures 6(c) and 6(d)).

## 4. Discussion

Gallbladder cancer is a pathogenic malignancy, affecting 2.5 per 100,000 people [18, 19]. lncRNA HOXA-AS2 has been found to be aberrantly expressed in a variety of human tumor tissues and cells (Supplementary Figure 1). HOXA-AS2 was highly expressed in gallbladder tumor tissues and cells. The molecular occurrence and progression of tumors is an extremely complex problem, in which cell cycle disturbance and epithelial-mesenchymal transition are common features of many types of human malignant tumor cells. Cell cycle disorders lead to uncontrolled cell proliferation, which greatly enhances the ability of cells to proliferate, while epithelial-mesenchymal transition makes cells lose contact inhibition and can move around [20, 21]. Epithelial-mesenchymal transition (EMT) also plays a significant role in the migration of gallbladder cancer cells. On the basis of this study, whether EMT is involved in the regulation of AS2 on the migration of gallbladder cancer cells can be further explored in the future. Our study confirmed that reducing HOXA-AS2 expression attenuated tumor cell proliferation in gallbladder cancer cell lines by the results of MTT assay and clone formation assay. The results of in vitro cell function experiments showed that interfering with the expression of HOXA-AS2 could inhibit the speed of in vitro migration of gallbladder cancer cells and the ability to invade other cells. At the same time, Western blot analysis of the expression of regulatory factors related to cell cycle and metastasis further confirmed that downregulation of HOXA-AS2 inhibited the migration of gallbladder cancer cells, but P21 protein and CyclinD1 protein were related to cell cycle. This may be related to the antiapoptotic effect of P21 protein [22–24]. Therefore, in this study, we explored the effect of HOXA-AS2 on cell cycle by detecting the expression of P21 protein and CyclinD1 protein.

Usually, lncRNAs and microRNAs (miRNAs) work together to regulate each other. We found that miR-6867-5p may be targeted by lncRNA HOXA-AS2 through bioinformatics tools. miR-6867-5p is a newly discovered miRNA with less research in the field of cancer. miR-6867-5p was found to be important in promoting angiogenesis under hypoxic conditions [25]. In this study, the expression of lncRNA HOXA-AS2 and miR-6867-5p strongly proves that lncRNA HOXA-AS2 and miR-6867-5p interact in the development of gallbladder cancer.

According to the prediction of the related software, YAP1 was predicted to be the target of miR-6867-5p, which was also confirmed by the detection of the luciferase reporter gene. The core components of signal transduction, Lats1/2, and Mst1/2, are often downregulated in some tumors due to the hypermethylation of their promoters, thereby promoting the malignant transformation of tumors [26]. Yes-associated proteins (YAPs) typically regulate signal transduction and gene transcription in cells [27, 28]. YAP is generally highly expressed in many tumors, and its nuclear activity is significantly enhanced, thereby inducing tumor progression [29–31]. lncRNA GAS5 interacts with YAP1 phosphorylation and degradation to inhibit rectal cancer progression [32]. However, how the Hippo-Yap signaling pathway plays its regulatory role in gallbladder cancer remains to be further clarified. The effect of lncRNA HOXA-AS2 on gallbladder cancer cells may affect the downregulation of miR-6867-5p and further affect the role of YAP1. In our study, YAP1 was first investigated in HOXA-AS2-knockdown gallbladder cancer cells. We found that YAP1 was downregulated in miR-6867-5p inhibitor and lncRNA HOXA-AS2 unsilenced groups, and upregulated in HOXA-AS2 knockout cells, indicating a regulatory relationship between lncRNA HOXA-AS2-miR-6867-5p-YAP1. In addition, unphosphorylated YAP is an active form, but YAP itself is a transcriptional coactivator that cannot bind DNA, so it needs to combine with other transcription factors such as TEAD1-4 to jointly initiate the transcription of downstream genes [33, 34]. Studies have shown that the downregulation of lncRNA HOXA-AS2 inhibits the phosphorylation of Yap/TAZ coactivator and its cytoplasmic retention, promotes its nuclear translocation, and promotes its function as a transcriptional coactivator, thereby promoting the expression of TEAD1-4 transcription factors. Activation. luciferase gene activity assay solution proves this. Our study also confirmed that after adding a Hippo pathway agonist, the invasive ability of gallbladder cancer cells with knockout of HOXA-AS2 were still lower. It is further proved that the Hippo signaling pathway-related regulates.

## 5. Conclusion

In conclusion, HOXA-AS2 may further regulate the occurrence and development of gallbladder cancer by regulating the miR-6867-5p-Yap pathway. HOXA-AS2 has important research significance in the study of potential diagnostic and therapeutic targets for gallbladder cancer. Due to the abnormal expression of HOXA-AS2 in hepatocellular carcinoma and other malignant tumors, the conclusion of this study may also be applicable to other tumor tissues. Subsequent experiments could also use the genes in this study to explore more cancer treatments.

## Data Availability

The datasets used and/or analyzed during the current study are available from the corresponding author upon reasonable request.

## Supplementary Materials

Supplementary Materials Supplemental Figure 1: TCGA analysis of HOXA-AS2 expression in different disease samples.
