# Supplementary material for: Effects of Long Noncoding RNA HOXA-AS2 on the Proliferation and Migration of Gallbladder Cancer Cells
Source: J Oncol. 2022 Oct 17;2022:6051512. doi: 10.1155/2022/6051512 (PMC9592229; doi:10.1155/2022/6051512)
Supplement: Supplementary Materials — Supplemental Figure 1: TCGA analysis of HOXA-AS2 expression in different disease samples. [file 6051512.f1.docx]

Supplemental Figure 1. TCGA analysis of HOXA-AS2 expression in different disease samples.
